# Case Log Trends of Graduating Obstetrics and Gynecology Residents Between 2018 and 2024

**DOI:** 10.1097/og9.0000000000000162

**Published:** 2026-03-19

**Authors:** Merima Ruhotina, Fei Cai, R. Nicholas Burns

**Affiliations:** Department of Obstetrics, Gynecology and Reproductive Sciences, University of Vermont, Burlington, Vermont; Department of Obstetrics and Gynecology, Division of Perinatology, Oregon Health & Science University, Portland, Oregon; and Department of Obstetrics and Gynecology, Division of Maternal Fetal Medicine, University of Texas Southwestern Medical Center, Dallas, Texas.

## Abstract

**OBJECTIVE:**

The aim of this study was to evaluate logged operative experience of obstetrics and gynecology residents in the United States between 2018 and 2024.

**METHODS:**

This was a retrospective analysis of publicly available Accreditation Council for Graduate Medical Education case log summary statistics of graduating obstetrics and gynecology residents. National mean case volumes per resident were summarized. Changes in operative experience between 2018 and 2024 classes were described with absolute and percent differences. Longitudinal trends across academic years were evaluated with Spearman rank correlation. To assess distributional changes in hysterectomies and operative vaginal deliveries, selected national percentiles (10th, 50th, and 90th) were additionally evaluated.

**RESULTS:**

Across multiple procedures, mean operative experience declined between 2018 and 2024, with the largest relative decreases observed in abdominal hysterectomy (−35.3%) and operative vaginal deliveries (−15.4%). Strong monotonic downward trends were observed over time for these procedures. In contrast, laparoscopic hysterectomy experience increased steadily across academic years. Percentile-based analyses showed parallel shifts at the 10th, 50th, and 90th percentiles for several procedures.

**CONCLUSION:**

Operative experience between 2018 and 2024 changed among graduating obstetrics and gynecology residents, with declining exposure in several core procedures and increased experience noted with laparoscopic hysterectomy. These findings highlight the importance of ongoing evaluation of training exposure in the context of national discussions regarding resident surgical readiness at the end of training.

## Abstract 2

Resident case logs demonstrate changes in case volume, including operative deliveries and hysterectomies; trends highlight a potential need to rethink surgical competency assessment.

The Accreditation Council for Graduate Medical Education (ACGME) is responsible for setting standards for U.S. residency training. This includes specifying a minimum surgical experience benchmark by specialty and procedure type.^1^ The ACGME reports annual summary statistics of surgical case logs of graduating residents from all accredited residency programs in the United States.

Over the past decade, shifts have been noted in obstetric and gynecologic surgical in-training case volumes. The most recent and comprehensive analyses evaluated case log trends through the 2017–2018 academic year. They showed an overall decreasing number of gynecologic cases per resident over time, specifically in total abdominal hysterectomies.^2,3^ The decrease in case numbers has also been observed in other surgical specialties.^4–9^ In addition to these preexisting trends, beginning in December 2019 the coronavirus disease 2019 (COVID-19) pandemic placed considerable strain on hospital systems, leading to major shifts in operations such as resource allocations and temporary suspensions of elective surgeries during the initial response. These disruptions may have had implications on case volumes and clinical exposure for residents and fellows in surgical specialties.^10^

No studies to date have evaluated the most recent case log data encompassing the years affected by the COVID-19 pandemic in obstetrics and gynecology. Therefore, the objective of this study is to characterize recent trends in reported gynecologic and obstetric case volumes among obstetrics and gynecology residents between 2018 and 2024 using national statistics from the ACGME. We hypothesize that surgical experiences are significantly different between residents who graduated at the beginning and end of this cohort.

## METHODS

Case log data were obtained from the publicly available ACGME Accreditation Data System Case Log Reports (https://apps.acgme.org/ads/Public/Reports/Report/25). These reports contain aggregate statistics on surgical and obstetrics case logs of graduating obstetrics and gynecology residents from all accredited programs in the United States. The ACGME reports the national resident average, median, SD, minimum, and maximum for each case type, in roles as both surgeon and teaching assistant. In addition, the ACGME reports case numbers at the 10th, 15th, 30th, 50th, 70th, 90th, and 95th percentiles by role for each procedure. The ACGME does not report any additional information beyond these summary statistics. Residents receive credit toward case minimums if they perform the procedure as the primary surgeon or as a senior teaching assistant.

For this study, both obstetric and gynecologic procedures were analyzed. The obstetrics cases include spontaneous vaginal deliveries, cesarean deliveries, operative vaginal deliveries, and obstetric ultrasound. Operative vaginal deliveries were further detailed into forceps-assisted vaginal deliveries and vacuum-assisted vaginal deliveries. The gynecologic procedures include total hysterectomies, operative hysteroscopy, other laparoscopies, transvaginal ultrasound, abortion, cystoscopy, incontinence and pelvic floor procedures, total invasive cancer, and total robotic procedures. Total hysterectomies were further delineated into total abdominal hysterectomies (TAHs), total laparoscopic hysterectomies (TLHs), and vaginal hysterectomies, including laparoscopic-assisted vaginal hysterectomy. Robot-assisted total hysterectomies were counted as TLHs. Case types included in the counts for incontinence and pelvic floor procedures and invasive cancer procedures have been previously described.^2^

Data were analyzed from the graduation years of 2018 through 2024. We selected these years for analysis because the 2018 graduates represent a cohort who completed training before the COVID-19 pandemic and the last year these data were analyzed in the literature.^2^ The 2024 graduates completed the entirety of their training during or after the pandemic and are the latest statistics available from the ACGME. This project was deemed exempt by the Oregon Health & Science University IRB.

Statistical analysis was performed with STATA 19.5. To assess the relationship between graduation year and case volume over time, Spearman rank correlations were calculated for average case volume and selected percentiles (10th, 50th or median, and 90th). Given the small number of time points, Spearman correlation coefficients (ρs) were interpreted descriptively, with emphasis on direction and consistency rather than formal hypothesis testing. Values of ρ>0.75 or ρ<−0.75 were considered to represent strong trends indicating consistent positive or negative directionality in case volume, respectively. Trend lines were created to visualize changes by year at select percentiles of case experience.

## RESULTS

During the study period, 9,065 residents graduated from obstetrics and gynecology residencies and submitted case log information to the ACGME. The annual number of total graduating obstetrics and gynecology residents at ACGME-accredited institutions increased from 1,277 in 2018 to 1,469 in 2024, an increase of 192 residents (15.0%).

Comparing residency graduates' case logs from the class of 2018 with those from the class of 2024, we found that the average number of obstetrics cases in which residents were listed as the primary surgeon or as a teaching assistant (“surgeon+teaching assistant”) decreased across most procedure types (Table 1 including teaching assistant numbers and Table 2 excluding them). Strong, monotonic downward trends in mean experience at the surgeon+teaching assistant level were noted for operative vaginal deliveries overall (ρ=−1.00) and for forceps-assisted vaginal deliveries (ρ=−0.96) and vacuum-assisted vaginal deliveries (ρ=−1.00) individually.

**Table 1.** Trends and Magnitude of Change in Mean Surgeon Plus Teaching Assistant–Level Case Experience, 2018–2024

	Case Type	2018 Mean±SD	2024 Mean±SD	Absolute Change (% Change)	Spearman ρ (2018–2024)
Obstetrics	SVD	283.9±71.6	285.4±74.7	1.5 (0.5)	0.61
	CD	233±71.3	224.9±63.5	−8.1 (−3.5)	−0.75
	OVD	23.4±11.6	19.8±8.0	−3.6 (−15.4)	−1.00
	FAVD	7.0±8.3	4.7±5.7	−2.3 (−32.9)	−0.96
	VAVD	16.4±10.0	15.1±7.4	−1.3 (−7.9)	−1.00
	OBUS	144.5±113.0	118.9±107.9	−25.6 (−17.7)	−0.39
Gynecology	TAH	42.5±13.3	27.5±11.4	−15 (−35.3)	−1.00
	VH	23.9±10.8	19.0±7.2	−4.9 (−20.5)	−0.96
	TLH	49.7±23.5	69.8±26.0	20.1 (40.4)	1.00
	Total Hysts	116.1±32.0	116.3±31.0	0.2 (0.2)	0.18
	Hysteroscopy	79.4±32.9	77.1±31.5	−2.3 (−2.9)	−0.61
	Laparoscopy	107.2±38.6	105.1±37.4	−2.1 (−2.0)	−0.43
	TVUS	81.0±44.9	69.1±37.8	−11.9 (−14.7)	−0.96
	Abortion	46.2±28.4	46.2±29.0	0 (0)	−0.58
	Cystoscopy	51.6±30.4	53.2±30.3	1.6 (3.1%)	0.36
	ISPF	57.3±35.2	45.9±24.8	−11.4 (−19.9)	−0.96
	Invasive cancer	57.8±33.1	46.5±24.2	−11.3 (−19.6)	−1.00
	Robotic	33.6±35.0	49±35.6	15.4 (45.8)	0.77

SVD, spontaneous vaginal delivery; CD, cesarean delivery; OVD, operative vaginal delivery; FAVD, forceps-assisted vaginal delivery; VAVD, vacuum-assisted vaginal delivery; OBUS, obstetric ultrasound; TAH, total abdominal hysterectomy; VH, vaginal hysterectomy; TLH, total laparoscopic hysterectomy; Total Hysts, total hysterectomies; TVUS, transvaginal ultrasound; ISPF, incontinence and pelvic floor procedures; Robotic, total robotic procedures.

Spearman ρ reflects monotonic trends in national mean case volume across graduating years 2018–2024; values closer to ±1 indicate greater consistency of directional change.

N=1,277 graduates in 2018 and 1,469 graduates in 2024.

**Table 2.** Trends and Magnitude of Change in Mean Surgeon-Level Case Experience, 2018–2024

	Case Type	2018 Mean±SD	2024 Mean±SD	Absolute Change (% Change)	Spearman ρ (2018–2024)
Obstetric	SVD	254.3±69.2	238.7±66.9	−15.6 (−6.1)	−0.96
	CD	209.2±71.6	194.2±61.5	−15.0 (−7.1)	−0.85
	OVD	21.7±11.6	17.7±7.9	−4.0 (−18.4)	−1.00
	FAVD	6.5±7.9	4.3±5.2	−2.2 (−33.8)	−0.96
	VAVD	15.2±9.8	13.4±7.2	−1.8 (−11.8)	−1.00
	OBUS	141.4±111.2	113.8±101.9	−27.6 (−19.5)	0.11
Gynecology	TAH	40±12.9	26.2±10.8	−13.8 (−34.5)	−1.00
	VH	22.8±10.6	18.1±7.1	−4.7 (−20.6)	−0.96
	TLH	47.7±22.9	67.2±25.4	19.5 (40.9)	1.00
	Total Hysts	110.4±31.3	111.4±30.4	1.0 (0.9)	0.14
	Hysteroscopy	76.5±32.7	73.3±31.7	−3.2 (−4.2)	−0.89
	Laparoscopy	102±36.4	99.4±35.5	−2.6 (−3.2)	−0.57
	TVUS	79.7±44.6	67.2±37.9	−12.5 (−15.7)	−0.96
	Abortion	44.6±28.5	43.9±28.8	−0.7 (−1.6)	−0.68
	Cystoscopy	49.6±28.7	51.2±28.5	1.6 (3.2)	0.57
	ISPF	55.7±34.3	44.5±24.0	−11.2 (−20.1)	−0.94
	Invasive cancer	56±32.1	45.2±23.6	−10.8 (−19.3)	−1.00
	Robotic	32.9±34.5	47.4±34.3	14.5 (44.1)	0.77

SVD, spontaneous vaginal delivery; CD, cesarean delivery; OVD, operative vaginal delivery; FAVD, forceps-assisted vaginal delivery; VAVD, vacuum-assisted vaginal delivery; OBUS, obstetric ultrasound; TAH, total abdominal hysterectomy; VH, vaginal hysterectomy; TLH, total laparoscopic hysterectomy; Total Hysts, total hysterectomies; TVUS, transvaginal ultrasound; ISPF, incontinence and pelvic floor procedures; Robotic, total robotic procedures.

Spearman ρ reflects monotonic trends in national mean case volume across graduating years 2018–2024; values closer to ±1 indicate greater consistency of directional change.

N=1,277 graduates in 2018 and 1,469 graduates in 2024.

With regard to gynecologic cases, the average 2018 graduate likewise had more exposure than a 2024 graduate for most procedures (Tables 1 and 2). Total hysterectomies between the two graduating classes were overall similar, but experiences by individual modality were different. The 2024 graduates completed more TLHs but fewer TAHs and vaginal hysterectomies. Strong monotonic downward trends in mean experience were noted for gynecologic cases at the surgeon+teaching assistant level for TAH (ρ=−1.00), vaginal hysterectomy (ρ=−0.96), transvaginal ultrasound (ρ=−0.96), incontinence and pelvic floor procedures (ρ=−0.96), and invasive cancer procedures (ρ=−1.00). In contrast, TLHs (ρ=1.00) and robotic cases (ρ=0.77) had strong monotonic positive trends in mean case volume (Table 1).

**Fig. 1.**
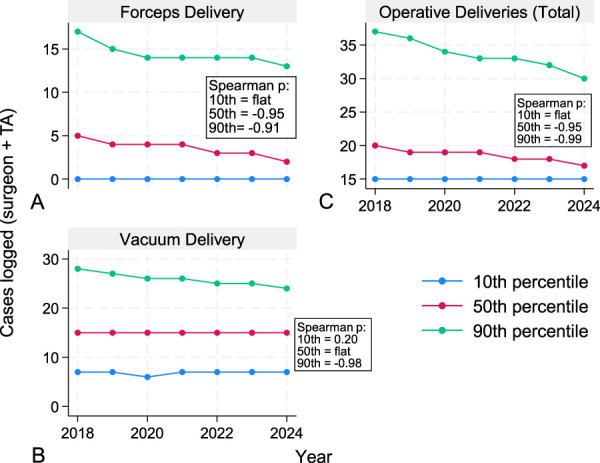
Graduating residents' operative delivery case log experience over time. Line graphs demonstrate resident case log volume for operative deliveries at varying percentiles of experience. Forceps delivery **(A)**, vacuum delivery **(B)**, and total operative deliveries **(C)**. *Year* represents the year in which the resident graduated from residency. *Spearman ρ* reflects monotonic trends in case volume at the percentile of relevant experience across graduating years 2018–2024; *values closer to ±1* indicate greater consistency of directional change. TA, teaching assistant. Graph created with Strata.

**Fig. 2.**
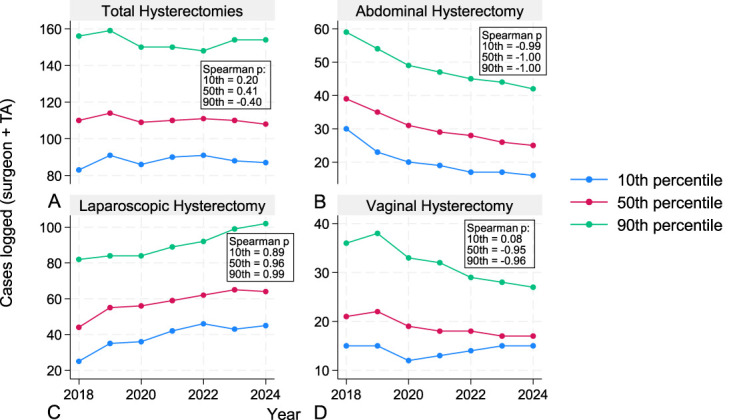
Graduating residents' hysterectomy case log experience over time. Line graphs demonstrate resident case log volume for hysterectomies at varying percentiles of experience. Total hysterectomies (all modalities) **(A)**, abdominal hysterectomy **(B)**, laparoscopic hysterectomy (including robotic) **(C)**, and vaginal hysterectomy **(D)**. *Year* represents the year in which the resident graduated from residency. *Spearman ρ* reflects monotonic trends in case volume at the percentile of relevant experience across graduating years 2018–2024; *values closer to ±1* indicate greater consistency of directional change. TA, teaching assistant. Graph created with Strata.

We further evaluated trends at the 10th, 50th, and 90th percentiles for surgeon+teaching assistant case experience of operative deliveries and hysterectomies. We performed these analyses to better assess whether resident case experience changes were globally appreciated or skewed toward high- or low-volume residents. For operative vaginal deliveries, strong negative monotonic trends were observed at the 90th percentile across both modalities and at the 50th percentile for forceps-assisted vaginal deliveries and total operative vaginal deliveries (Fig. 1). For hysterectomy, although no strong trend was observed for total numbers, strong negative monotonic trends were observed for TAH across all percentiles and the 50th and 90th percentiles for vaginal hysterectomy (Fig. 2). In contrast, TLH was observed to have a strong monotonic upward trend across all percentiles (Fig. 2).

*Ruhotina. Obstetrics and Gynecology Resident Case Log Trends, 2018-2024. O&G Open 2026*.

*Ruhotina. Obstetrics and Gynecology Resident Case Log Trends, 2018-2024. O&G Open 2026*.

The comparison of the 2018 experience percentiles of selected procedures and 2024 percentiles is presented in Table 3. For instance, 2024 graduates who logged 17 operative vaginal deliveries were in the 50th percentile, but in 2018, 17 operative vaginal deliveries was the 30th percentile. Similarly, 2024 graduates who logged 25 abdominal hysterectomies were in the 50th percentile, but in 2018, this number was below the 10th percentile. In contrast, 2024 graduates who logged 64 TLHs were in the 50th percentile, which was above the 70th percentile in 2018. Appendix 1 (available online at http://links.lww.com/AOG/E591) shows experience comparisons by graduation year across all procedure types.

**Table 3.** Estimated Percentile of Sample Case Experience, 2018 Compared With 2024

Procedure	Cases Logged (n)	Experience Percentile or Range, 2018 Graduate (%)	Experience Percentile or Range, 2024 Graduate (%)
OVD	17	30	50
	20	50	70
	25	70	70–90
TAH	20	Less than 10	30
	25	Less than 10	50
	30	10	70
TLH	45	50	10
	64	70–90	50
VH	15	10	10–30
	20	50	70

OVD, operative vaginal delivery; TAH, total abdominal hysterectomy; TLH, total laparoscopic hysterectomy; VH, vaginal hysterectomy.

Case count represents experience at the surgeon+teaching assistant level.

## DISCUSSION

From 2018 to 2024, resident surgical experiences logged in the ACGME system changed. In obstetrics, average operative delivery volume declined. This decline was also notable at the upper percentiles of experience. In gynecology, total hysterectomy counts remained stable, but case mix shifted toward laparoscopic approaches with concomitant decreases in abdominal and vaginal hysterectomies. Exposure to incontinence and pelvic floor procedures and invasive cancer procedures also demonstrated strong downward trends.

Our findings extend prepandemic trends showing declines in operative deliveries, vaginal hysterectomies, and abdominal hysterectomies and increases in laparoscopic hysterectomies.^2,3^ Although the pandemic may have played a role in some of the observed changes, it is important to recognize that our data do not have the ability to determine causation. Multiple factors likely contribute to these observations, including but not limited to rising trainee numbers, improved medical management, subspecialist involvement or referral patterns, and evolving surgical practices. Parallel concerns about declining or shifting operative exposure have been reported in multiple surgical specialties.^4–9^

The ACGME case log data are the primary national source of surgical volume of trainees. The summary findings are used for program benchmarking and accreditation decisions by the organization. At individual programs, complete case logs are frequently a graduation requirement. However, the data provide a limited view of training. The ACGME case minimums represent a minimum threshold for acceptable exposure but lack validation for resident competency. The summary statistics lack granularity by program, region, or resident characteristics. At the data entry level, case log inaccuracies are also possible. The data depend on manual entry from residents themselves and their interpretation of involvement in the case.^11,12^

Despite the limitations, case log summary statistics can raise important training concerns. The recent threshold increase for TAH from 15 to 20 minimum cases illustrates a potential misalignment between expectations and current resident exposure. If this TAH standard were applied to the class of 2024, more than 30% would be considered to have insufficient experience (Appendix 1, http://links.lww.com/AOG/E591). In the absence of evidence linking specific case volume thresholds to procedural competency, raising minimums may create stress for trainees and programs without advancing meaningful educational goals.

Advancing the patient- and education-centered mutual goal of surgically competent resident graduates in the face of declining or shifting case volumes will require innovations in teaching and assessment. The rise of minimally invasive surgery has accompanied standardized skills programs such as the Fundamentals of Laparoscopic Surgery and Essentials in Minimally Invasive Gynecologic Surgery to help formalize technical training.^13,14^ Comparable programs for open or vaginal surgery could similarly teach and preserve core skills. Workplace-based assessment systems can measure real-world operative performance and provide evidence for the relationship between case volume and skill acquisition.^15–20^ Surgical coaching models may enhance reflection and continuous improvement at scale.^21–23^ Some surgical specialty certification boards have begun incorporating surgical portfolios and early-career coaching programs after the completion of residency training to advance similar goals.^24,25^

Finally, broader structural innovations are being debated, including the extension of residency to 5 years, early subspecialty “tracking,” and the ACGME Flexibility in Training pilot, which tailors case exposure to future career paths.^26–30^ It is unclear which, if any, of these represent the best path forward. However, maintaining static training structures amid changing surgical case numbers is unlikely to meet the needs of trainees, patients, or the future of obstetrics and gynecology. Continued evaluation of how surgical competence is developed, assessed, and supported, moving beyond case logs alone, will be essential to understanding how to mitigate these changes in surgical volume.
